# Summary Visualizations of Gene Ontology Terms With GO-Figure!

**DOI:** 10.3389/fbinf.2021.638255

**Published:** 2021-04-01

**Authors:** Maarten J. M. F. Reijnders, Robert M. Waterhouse

**Affiliations:** Department of Ecology and Evolution, Swiss Institute of Bioinformatics, University of Lausanne, Lausanne, Switzerland

**Keywords:** functional genomics, python software, redundancy reduction, semantic similarity, GO term enrichment

## Abstract

The Gene Ontology (GO) is a cornerstone of functional genomics research that drives discoveries through knowledge-informed computational analysis of biological data from large-scale assays. Key to this success is how the GO can be used to support hypotheses or conclusions about the biology or evolution of a study system by identifying annotated functions that are overrepresented in subsets of genes of interest. Graphical visualizations of such GO term enrichment results are critical to aid interpretation and avoid biases by presenting researchers with intuitive visual data summaries. Amongst current visualization tools and resources there is a lack of standalone open-source software solutions that facilitate explorations of key features of multiple lists of GO terms. To address this we developed GO-Figure!, an open-source Python software for producing user-customisable semantic similarity scatterplots of redundancy-reduced GO term lists. The lists are simplified by grouping together terms with similar functions using their quantified information contents and semantic similarities, with user-control over grouping thresholds. Representatives are then selected for plotting in two-dimensional semantic space where similar terms are placed closer to each other on the scatterplot, with an array of user-customisable graphical attributes. GO-Figure! offers a simple solution for command-line plotting of informative summary visualizations of lists of GO terms, designed to support exploratory data analyses and dataset comparisons.

## Introduction

Since its foundation more than two decades ago, the Gene Ontology (GO) knowledgebase has developed into the world's gold-standard for comprehensively describing gene functions (Ashburner et al., 2000; The Gene Ontology Consortium, 2019). GO terms capture biological knowledge in three formalized ontologies (Biological Process, Molecular Function, and Cellular Component) that are hierarchical with “child” terms being more specific than their “parent” terms. Being both human-readable and machine-readable, and capturing these relationships in a directed acyclic graph (DAG), make the GO a powerful tool for computational analyses of results from large-scale molecular biology experiments and genome-wide assays (Dessimoz and Škunca, 2017). Typically, these results identify subsets of genes of interest, e.g., with increased or decreased expression levels under different conditions or treatments, or with high or low population-level genetic variation. GO enrichment analysis is subsequently performed to determine what is special or different about the subset of genes with respect to their associated processes, functions, or components. These results can then be used to support or refute hypotheses, inferences, or conclusions about the biology or evolution of the study system. Enrichment analysis is generally performed by testing for statistical overrepresentation of GO terms annotated to genes from the subset of interest, using enrichment tools such as the TopGO (Alexa and Rahnenfuhrer, 2020) or GOStats (Falcon and Gentleman, 2007) R packages. Test results from various enrichment tools are usually presented as lists of terms with their associated probabilities (*p*-values), possibly also with the counts of annotated genes in the foreground (subset of interest), or ranks and *p*-values from different classes of statistical tests. While comprehensive, these lists can be difficult to interpret and may be subject to biases when attempting to summarize the key results, mainly because they do not present information on the relationships amongst the listed terms. Graphical visualizations of enrichment results have therefore been developed to aid interpretation and present visual summaries (Supek and Škunca, 2017). TopGO can provide views of how the most significant terms are distributed over the GO DAG, and results from TopGO and GOStats can be explored using R graphics functions (R Core Team, 2021). However, online web-server applications such as AmiGO (Carbon et al., 2009), GOrilla (Eden et al., 2009), and REVIGO (Supek et al., 2011), are much more widely used for visualizing results and producing summary figures for publications. Of these, REVIGO summaries are particularly popular as they simplify lists by grouping together terms with similar functions, using semantic similarities. Graphical summaries of the redundancy-reduced results can be produced as scatterplots that place more semantically similar terms closer to each other on the plot. Several options are provided that allow for interactive user-customization of the graphics, and plotting R scripts can be downloaded for further customization.

Despite the importance of using up-to-date versions of gene-term annotations and the core GO (Wadi et al., 2016), web-server applications often do not keep pace with the evolution of the ontology and the annotations. This can lead to inconsistent or erroneous output summary visualizations generated from non-version-matched input lists of enriched GO terms. Additionally, web-server applications generally require input lists of terms to be individually uploaded by the user for each analysis they wish to conduct, making it difficult to investigate the main features of multiple lists. Exploratory data analysis is greatly facilitated by graphical data summaries (Lee et al., 2020), so being able to visually compare results from multiple lists is particularly important in this context. For example, using different software, parameters, normalisations, or filters to quantify differential gene expression will impact the results of gene set enrichment analyses. Alternatively, if gene lists are defined by clustering procedures, varying the input features, distance functions, or clustering algorithms will impact cluster membership and consequently the results of enrichment analyses. Beyond data exploration, visualizing similarities or differences amongst enriched GO terms from multiple lists can support interpretations or conclusions drawn from comparing results from different tissues, life-stages, conditions, treatments, or even different species or populations.

To enable explorations and comparisons of graphical summaries of lists of GO terms we developed GO-Figure!, an open-source Python software for producing user-customisable semantic similarity scatterplots. Using the latest core GO, or user-defined versions to match their enrichment analysis results, similarity scores are used to group redundant terms from which a representative is selected for plotting in two-dimensional semantic space, with user-control over grouping thresholds and scatterplot graphical attributes. GO-Figure! offers a simple solution for command-line plotting of informative data summary visualizations to support exploratory data analyses and dataset comparisons.

## Materials and Methods

GO-Figure! is a Python software designed to facilitate the graphical visualization of results from GO term enrichment analyses. User-provided input lists are first assessed to remove obsolete GO term identifiers and update them with replacement identifiers if defined in the core GO. Redundancies in the list of significantly enriched terms are minimized according to user-specified thresholds using pairwise semantic similarities to group similar terms together and subsequently select a representative for the group. These redundancy-reduced term lists are then visualized in two-dimensional semantic space where more functionally similar terms are placed closer to each other on the scatterplot.

### Redundancy-Reduced GO Term Lists

Redundancy reduction aims to identify similar GO terms and group them together in order to reduce the total number of terms and simplify the summary visualization. For a user-provided list of terms derived from the results of a GO term enrichment analysis such as those produced by TopGO or GOStats, pairwise semantic similarities are calculated with the commonly-used formula proposed by Lin (1998):


(1) $$Sim\left(GO_{i},GO_{j}\right)=\frac{2\;\cdot \;\max_{go\in S(go_{i},go_{j})}\{IC\left(go\right)\}}{IC\left(go_{i}\right)+IC\left(go_{j}\right)}$$

Here, *S* is the subset of GO terms shared between terms *i* and *j* after propagating up the GO DAG using the “is_a” and “part_of” relations. GO-based semantic similarities leverage the structure of the GO DAG to define a numerical value representing the closeness in meaning between any two terms (Pesquita, 2017). It can be likened to measuring the minimum number of steps across the graph required to connect two terms, weighted by how specific or general the terms are. The weights of GO term specificities derive from their information content (*IC*), which is the relative frequency of a GO term *i* compared to the total number of GO terms in the UniProt (The UniProt Consortium, 2019) Gene Ontology Annotation (GOA) database:


(2) $$\begin{array}{r} IC\left(GO_{i}\right)=\;-log\left(\frac{\left\{go:go_{i}\;\in GOA\right\}}{\left\{go:go\;\in GOA\right\}}\right) \end{array}$$

Each term is grouped with its most similar term if their pairwise semantic similarity score is higher than or equal to a user-adjustable threshold value. Higher thresholds result in smaller groups of terms that are often close together on the GO DAG while lower thresholds can produce larger groups of more distantly related terms (Supplementary Figure 1). One term is then selected as the representative of the group following the logical reasoning outlined below. If the most similar term is already part of another group, then the term is added to this group and a representative term is selected between the newly added term or the current representative of the group. This process is repeated for all terms provided in the list from the enrichment analysis, resulting in groups of terms represented by a single term, based on their semantic similarities, with user-control over the resolution of redundancy reduction and representative term selection. Terms with no other term above the semantic similarity score threshold remain as singletons on the scatterplot. Representative selection is based on the following stepwise logical reasoning:

- If one GO term is annotated to 5% or more of the proteins in the GOA database (i.e., it is a term with a broad interpretation), select the other more specific term. If both terms are annotated to 5% or more, proceed to step 4.
- If their *p*-values are substantially different, i.e., one GO term has a 50% higher *p*-value than the other, then select the term with the lower *p*-value.
- If one GO term is a parent term of the other term, select the parent term.
- If none of the above is true, select the first GO term as the representative (Python ordering of list objects results in a deterministic choice and therefore reproducible selections).

### Semantic Similarity Scatterplots

The redundancy-reduced lists of GO terms are summarized as circles plotted in two-dimensional semantic similarity space for each representative term, where more similar terms are plotted closer together on the x and y axes of the scatterplot (labeled semantic space X and Y). The pairwise semantic similarities for all representatives are used to generate the two-dimensional transformation using the multidimensional scaling algorithm from SciKit-Learn (Pedregosa et al., 2011). The two-dimensional transformation generates a solution that places terms with higher semantic similarities closer together on the scatterplot (Supplementary Figure 2). The average distances amongst terms within groups increases as the semantic similarity threshold is relaxed, and average distances between groups are substantially larger (Supplementary Figure 3). By default, the color of the circle indicates the user-provided significance (log10 *p*-value) of that GO term (the representative term), and the size indicates the numbers of terms being represented by that term (the number of terms in the group). Several options are provided to allow users to easily configure how colors and sizes are used in plotting. The scatterplots themselves are generated using the Seaborn (Waskom et al., 2020) and Matplotlib (Hunter, 2007) Python packages.

### Gene Ontology Datasets

The calculations of semantic similarities and information contents detailed above rely on GO datasets including the core ontology from the GO Knowledgebase in Open Biomedical Ontologies (OBO) format that includes all parent-child relationships, and the GOA database of GO annotations from UniProt. The importance of using up-to-date and version-matched gene-term annotations and the core GO used for enrichment analyses is often overlooked (Wadi et al., 2016). GO-Figure! is provided with up-to-date go.obo ontologies and precomputed GO term relations and information contents scores. User-provided input lists are checked for obsolete GO terms, which are updated if replacements are defined in the go.obo or discarded, with user notifications printed to a log file. As the core GO and the UniProt GOA database are regularly updated, support scripts and instructions are provided to compute the required GO term relations and information contents scores for user provided versions of the core GO and the UniProt GOA. To ensure that all terms present in the user-provided list can be assessed, it is recommended to use the same go.obo data version that was used for the GO term enrichment analysis, and the latest corresponding release of the GOA from UniProt. Versions used to produce the figures in this manuscript: go.obo data version 18-11-2020, goa_uniprot_all.gaf version 10-7-2020.

## Results

### Summary Visualizations With GO-Figure!

GO-Figure! is designed to respond to two major gaps amongst current tools and resources for building summary visualizations of lists of GO terms: (1) a lack of GO-version-aware standalone software solutions; and (2) a lack of solutions that facilitate exploratory data analyses and dataset comparisons. It implements community-recognized definitions of semantic similarity and information content, following simple term grouping rules, using an established multidimensional scaling algorithm to compute the two-dimensional semantic space, and popular Python packages for plotting the results. Together with up-to-date GO data, GO-Figure! brings these components together to offer users a flexible new approach for exploring their lists of GO terms, using different aggregation stringencies and plotting options to build summary visualizations that convey the key features of their data. It is provided as an open-source Python software, which is simple to run even for novice Python users and requires no additional programming skills unlike other software that require running R packages or using application programming interfaces. It checks the validity of user-supplied lists with up-to-date ontologies and annotations, and provides user-control over version matching. As a standalone software it also allows for the rapid production of graphical summaries for multiple datasets without the need to repeatedly upload lists to an online web-server. Summary visualizations are presented as scatterplots across two-dimensional semantic similarity space (see Materials and Methods), an intuitive data summary format popularized by REVIGO. Before plotting, semantic similarities and information contents are used to group similar GO terms and select a representative for each group, thereby achieving reduced redundancy of the displayed GO terms.

**Figure 1:**
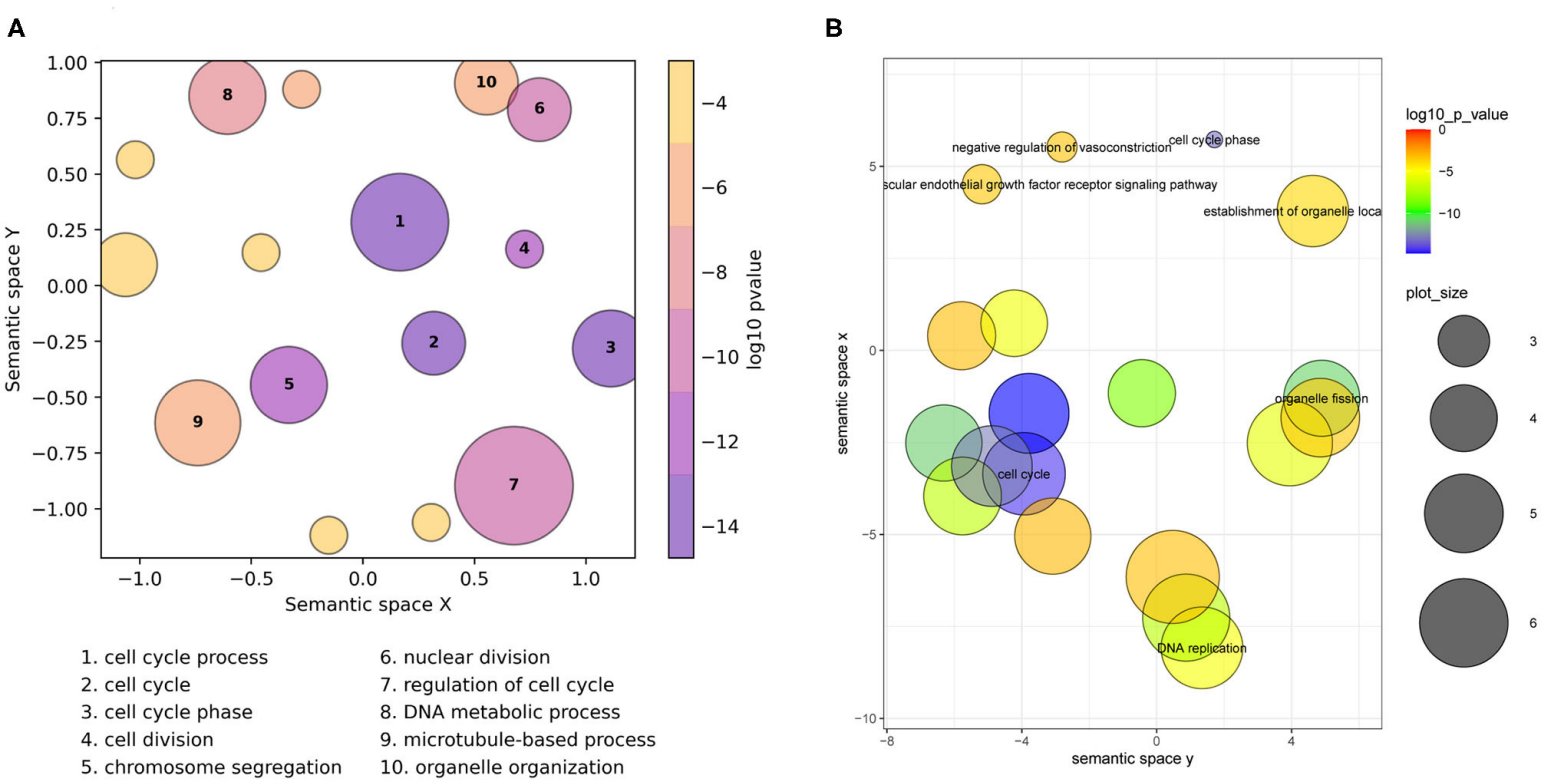
Semantic similarity scatterplots summarizing a list of enriched Gene Ontology (GO) terms from a breast cancer differential gene expression study. The scatterplots show GO terms as circles arranged such that those that are most similar in semantic space X and Y are placed nearest to each other. They show results employing default plotting options with **(A)** GO-Figure! generated using the Seaborn and Matplotlib Python packages, and **(B)** the REVIGO web-server generated with the provided R script using the ggplot2 and scales R packages. Both use Lin (1998) semantic similarities with a threshold of 0.7 for redundancy reduction. The input GO term list from the REVIGO website consists of 46 Biological Process terms from an enrichment analysis of data from Van't Veer et al. (2002). Both scatterplots show the significance obtained from the enrichment analysis using a gradient color palette (log10 *p*-value), and the sizes of the plotted circles are scaled by the number of GO terms they represent for GO-Figure! and by the GO term frequency for REVIGO. In **(A)** the 10 most significant terms are labeled with numbers and their descriptions are provided below the plot, in **(B)** the seven most significant terms are labeled with their descriptions. Full lists of terms and their groupings are provided in Supplementary Tables 1, 2.

On the scatterplot, terms are arranged such that those which are most similar in semantic space X and Y are placed nearest to each other (Figure 1, Supplementary Figure 2). Importantly, it is the relative positioning of the plotted terms that summarizes the key functional information. The axes themselves are the result of dimensionality reduction using the provided list, so they are list-specific and convey no particular functional meaning. By default, the *p*-value obtained from the enrichment analysis that generated the list is visualized using a gradient color palette, and the sizes of the plotted circles are scaled by the number of terms they represent. Figure 1A illustrates the basic functionality of GO-Figure! using an input list of 46 terms (biological processes) derived from data from Van't Veer et al. (2002), with term groupings listed in Supplementary Table 1. This list is provided as an example dataset on the REVIGO web-server generated by analyzing differentially expressed genes between breast cancer patients with fewer than 5 years to metastases and at least 5 years disease-free. Figure 1B shows the data summary produced selecting the Lin semantic similarity measure and otherwise using default settings on the REVIGO web-server for the same list of 46 terms, with term groupings listed in Supplementary Table 2.

GO-Figure! reduces redundancy to 16 representative terms, the 10 most significant are labeled on the scatterplot and their descriptions are shown below (Figure 1A). Six are singletons and the other ten represent the remaining 40 terms, the smallest group two similar terms, e.g., *organelle fission* + *nuclear division*; *cell cycle* + *mitotic cell cycle*; *cellular response to DNA damage stimulus* + *cellular response to stress*, or three similar terms, e.g., *chromosome segregation* + *sister chromatid segregation* + *mitotic sister chromatid segregation*; *G2 phase* + *cell cycle phase* + *mitotic G2 phase*, the largest groups 13 terms represented by *regulation of cell cycle* (Supplementary Table 1), and the grouped terms are usually close together on the GO DAG (Supplementary Figure 4). GO-Figure! recognizes one term identifier (GO:0007067) as obsolete and instead uses the replacement term identifier (GO:0000278) as defined by the latest core GO. Increasing the semantic similarity score threshold results in more (*n* = 44), smaller groups while decreasing the threshold produces fewer (*n* = 9), larger groups (Supplementary Table 3). Because less similar terms are progressively grouped together as the threshold is lowered, comparing results using different thresholds allows users to develop the most meaningful summary of their data.

REVIGO reduces redundancy to 19 representative terms, seven of which are labeled with their descriptions on the scatterplot (Figure 1B), employing the January 2017 version of the GO and the March 2017 version of the UniProt GOA provided online. Singletons make up 12 of the representatives, including four of the six GO-Figure! singletons. The remaining 34 GO terms are represented by seven groups, the smallest group two similar terms, *cytoskeleton organization* + *DNA conformation change*, or three similar terms, e.g., *spindle organization* + *microtubule polymerization or depolymerization* + *microtubule cytoskeleton organization*; *establishment of organelle localization* + *establishment of spindle localization* + *establishment of mitotic spindle localization*; *G2 phase* + *cell cycle phase* + *mitotic G2 phase*, and the largest groups 14 terms represented by *cell cycle process* (Supplementary Table 2).

The scatterplots reduce the full list of terms into visualizations that capture the main biological features from the results of differential gene expression analysis of breast cancer patients that did and did not develop metastases: dominated by processes linked to active cell proliferation. Qualitatively the main differences are in the membership and representative selection of the largest groups: the REVIGO group represented by *cell cycle process* contains core and regulatory processes while the GO-Figure! group represented by the same term collects organization and localization processes, with a separate group containing regulatory processes represented by *regulation of cell cycle* (labeled “7” in Figure 1A).

### Customizing Summary Visualizations

**Figure 2:**
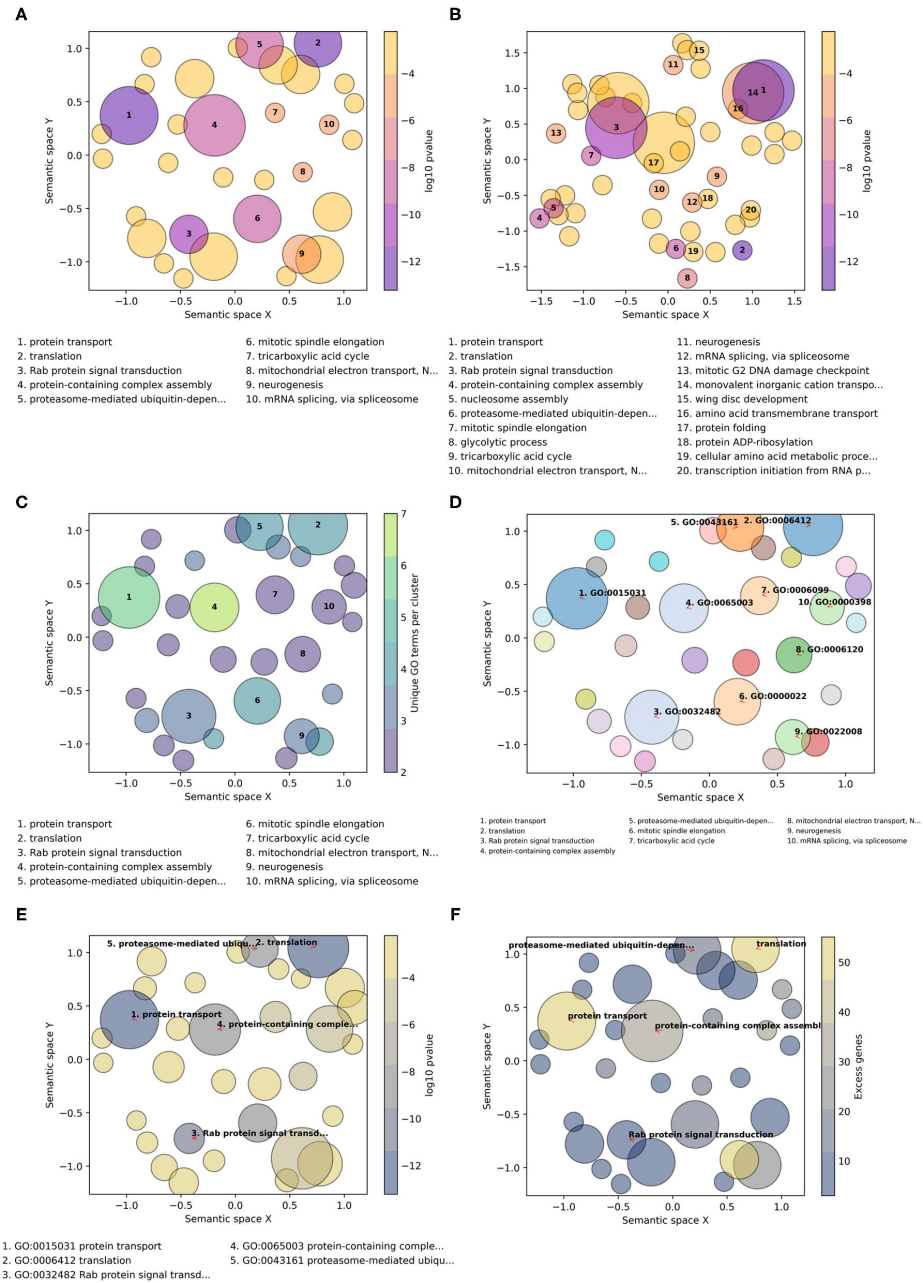
Example customizations of GO-Figure! summary visualizations. The input GO term list consists of 60 Biological Process terms from an enrichment analysis of genes with low protein sequence divergence rates from Neafsey et al. (2015). One term was recognized as obsolete and discarded, four others were updated with their replacement GO term identifiers. **(A)** Default plotting options, i.e., semantic similarity threshold of 0.5 and 10 numbered labels. **(B)** Increasing the semantic similarity threshold to 0.8 and plotting labels for the 20 most significant GO terms. **(C)** Using the color palette “viridis,” with semantic similarity threshold of 0.5, colors based on the number of GO terms being represented (members), and sizes based on *p*-values. **(D)** Color palette “tab20” with a unique color per circle, semantic similarity threshold of 0.5, three-column reduced font size legend for 10 GO term identifier labels with arrows, and sizes based on *p*-values. **(E)** Color palette “cividis” with colors based on *p*-values and sizes based on user-provided values (excess genes, i.e., the difference between the number of genes in the foreground annotated with the term and the expected number given the total number of genes annotated with the term), five descriptor labels limited to 25 characters, with GO term identifiers and descriptors in the legend. **(F)** Palette “cividis” with colors based on user-provided values of excess genes, sizes based on *p*-values, and five descriptor labels with no legend.

GO-Figure! allows for user-customization to tailor three main features of the resulting scatterplots: (1) color palettes and scaling; (2) management of GO term labels; and (3) incorporating user-provided additional data. This is achieved through a wide selection of command line options detailed in the software help and the user guide. A large choice of color schemes is made available through the full library of color brewer palettes provided by the Matplotlib Python package, e.g., the popular plasma or viridis palettes (Figure 2). By default, as shown in Figure 2A, circles are colored based on the user-provided *p*-values (log10-scaled) for each term, and size-scaled by the number of terms they represent (members, resulting from the redundancy reduction steps). Applying a semantic similarity score threshold of 0.5 results in 33 representatives (Figure 2A) and increasing it to 0.8 produces 54 groups (Figure 2B). Across a range of thresholds from 1.0 (all 59 terms) to 0.1 (11 groups), less similar terms are progressively grouped together as the threshold is lowered (Supplementary Table 4). Colors can instead be used to indicate the number of members, the frequency of the term in the GOA database (information content score), or simply a unique color per term. Similarly, *p*-values, members, or GOA frequency, can be used to size-scale the circles, or a fixed size can be applied.

The number of GO terms to be plotted depends on how many were supplied in the input list and on the stringency applied during redundancy reduction. To avoid cluttering the scatterplot with term descriptions, the most significant terms are labeled with numbers and their descriptions are printed below the scatterplot (Figures 2A–C). Command line options allow users to determine the numbers of terms to be labeled and manage the layouts of the descriptions (font sizes, number of columns, maximum character lengths of descriptors, etc.). The GO terms on the scatterplot can instead be labeled with their identifiers, or with their descriptors, using automatic label placement (Flyamer et al., 2020) to optimize readability (Figures 2D–F). This provides the user with the flexibility to easily increase or decrease the amount of in-plot labeling text in a standardized manner. The scatterplots in Figure 2 summarize a list of 60 Biological Process terms from an enrichment analysis of mosquito genes with the lowest protein sequence divergence rates (Neafsey et al., 2015). Such highly constrained genes are expected to be involved in widely conserved essential processes. The summary visualizations highlight core developmental functions such as neurogenesis, metabolic pathways such as the tricarboxylic acid cycle, and essential core biology such as translation and protein transport. Thus, their low levels of sequence divergence can be explained by the conserved processes in which they are involved.

The minimal user-provided input information required for building the summary visualizations is the input list of GO term identifiers with their corresponding probabilities resulting from enrichment testing (standard input). GO-Figure! is also able to process default files produced from enrichment testing with TopGO or GOStats, consisting of seven columns as detailed in the user guide. The values from the “Significant” (TopGO) and “Count” (GOStats) columns, which provide counts of the numbers of genes in the foreground set annotated with the term, can be used to determine plotting colors and sizes. Alternatively, users can prepare a “standard-plus” input consisting of the term identifiers, their corresponding probabilities, and a third column with a user-defined metric that can also be used to determine plotting colors and sizes (Figures 2E,F). For example, the total numbers of genes annotated with the term in the specific annotation dataset used during enrichment testing, rather than frequencies calculated from the entire UniProt GOA database.

The scatterplots aim to summarize information through redundancy reduction, hence only representative GO terms are shown for groups of similar terms. For user-reference, group membership information is provided in tab-delimited text files as part of the default output. With these details provided in the text output, and being able to explore the effects of different stringencies and plotting options, users are able to reach confident conclusions about the key biological features that characterize their GO term lists. For example, GO term enrichment analysis of genes in subcutaneous adipose tissue after sleep deprivation found hypermethylated genes to be associated with lipid response and cell differentiation pathways while hypomethylated genes were related to pathways such as lipid metabolism and regulation of DNA damage responses (Cedernaes et al., 2018). Each with more than 100 terms, visually comparing these lists and exploring their redundancy-reduced groupings (Supplementary Figures 5, 6, Supplementary Tables 5, 6) provides support for the study's conclusions, as well as suggesting that the hypermethylated genes are also associated with signaling processes and that roles in skeletal muscle wasting could also be a key feature of the hypomethylated genes.

Finally, for tracking provenance and facilitating reproducibility a log file is created to record the versions of GO-Figure!, the core GO, and the UniProt GOA database used, as well as the options and parameters used to produce the scatterplot, and to track any term identifiers that were either updated to correspond with the current version of the GO or that were discarded because they were found to be obsolete with no replacements. In addition, the Python data frames used to create the scatterplots are also saved as tab-delimited text files. This allows for users with more advanced experience of Matplotlib functions to further customize the summary visualizations, to integrate them into multi-panel figures, or to create interactive versions of the scatterplots.

## Conclusion

Recognizing the usefulness and popularity of semantic-similarity-based redundancy reduction for producing visualizations of GO term lists, GO-Figure! offers a simple solution for command-line plotting of informative graphical summaries. It addresses a lack amongst current visualization tools and resources of standalone open-source software solutions that are GO-version-aware and facilitate explorations and comparisons of multiple lists of GO terms. Summary visualizations of gene set functional annotations with GO-Figure! enable researchers to perform exploratory data analyses and multiple dataset comparisons to support hypotheses or conclusions about the biology or evolution of their study systems.

## Data Availability Statement

Publicly available datasets were analyzed in this study. This data can be found here: https://gitlab.com/evogenlab/GO-Figure.

## Author Contributions

MR and RW: conception and wrote the manuscript. MR: software development. All authors read and approved the manuscript.

## Conflict of Interest

The authors declare that the research was conducted in the absence of any commercial or financial relationships that could be construed as a potential conflict of interest.
