# Supplementary material for: Summary Visualizations of Gene Ontology Terms With GO-Figure!
Source: Front Bioinform. 2021 Apr 1;1:638255. doi: 10.3389/fbinf.2021.638255 (PMC9581009; doi:10.3389/fbinf.2021.638255)

Supplementary Material

Supplementary Tables

Supplementary Tables S1, S2, S3, S4, S5, and S6 are provided as an Excel file:
Supplementary_Tables.xlsx

Table S1. GO-Figure! summary data of input GO terms and their representatives after redundancy reduction as shown in Figure 1A. For each GO term columns present: representative GO term, member GO term, GO term description, p-value from enrichment testing, computed information content (IC), and computed frequency in the UniProt GOA.

Table S2. REVIGO summary data of input GO terms after redundancy reduction as obtained from the REVIGO web-server to produce Figure 1B, with the addition of a column to indicate the representative GO term selected during redundancy reduction.

Table S3. GO-Figure! GO term groupings with selected representatives for the Figure 1A GO term list using ten semantic similarity thresholds from 1.0 to 0.1. The most stringent threshold results in 44 groups while the least stringent produces just nine groups. The cells are coloured to show the generally progressive merging of terms and groups of terms into larger groups as the semantic similarity threshold is reduced.

Table S4. GO-Figure! GO term groupings with selected representatives for the Figure 2A GO term list using ten semantic similarity thresholds from 1.0 to 0.1. The most stringent threshold results in 49 groups while the least stringent produces just five groups. The cells are coloured to show the generally progressive merging of terms and groups of terms into larger groups as the semantic similarity threshold is reduced.

Table S5. GO-Figure! GO term groupings with selected representatives for the GO term list visualised in Figure S5, based on hypermethylated genes in subcutaneous adipose tissue after sleep deprivation.

Table S6. GO-Figure! GO term groupings with selected representatives for the GO term list visualised in Figure S6, based on hypomethylated genes in subcutaneous adipose tissue after sleep deprivation.

Supplementary Figures

Figure S1. Terms and groups of terms shown on a subset of the Gene Ontology Biological Process graph using the 59 terms from main text Figure 2. (**A**) Depicts groupings from Figure 2A, with 33 term representatives at a semantic similarity threshold of 0.5, zooming into parts of this graph in (**B-E**) for clarity. (**F**) Depicts groupings from Figure 2B, with 54 term representatives at a threshold of 0.8, zooming into the centre of this graph in (**G**) for clarity. Singleton terms are shown in light red, contrasting colours highlight 15 groups of terms in A-E and five pairs of terms in F-G. The graph was drawn using the GOView web-server from http://www.webgestalt.org/ with groups of terms subsequently manually highlighted with different colours.

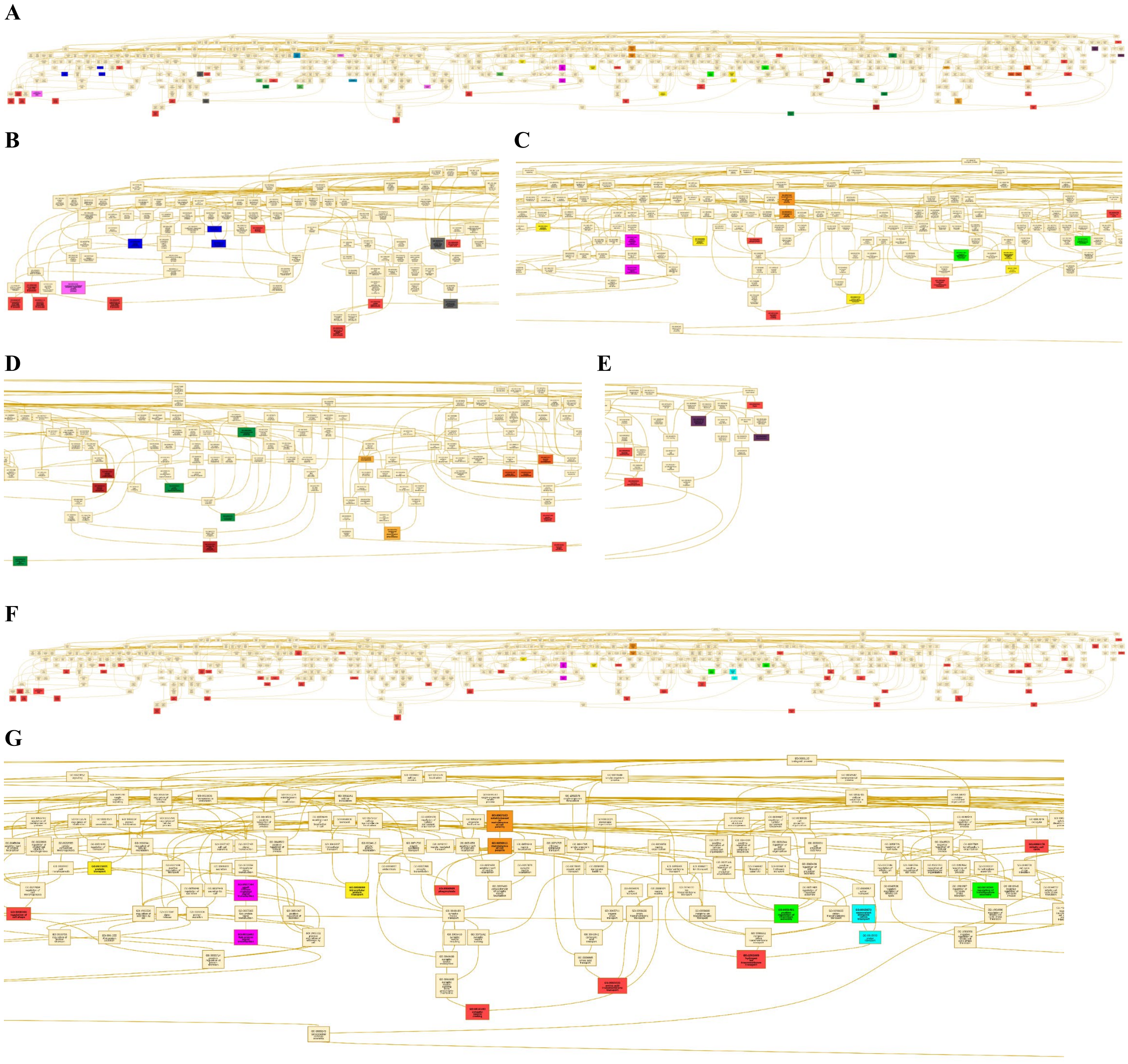

Figure S2. Semantic similarities versus scatterplot distances for all pairs of Gene Ontology terms shown in the main text (**A**) Figure 1A, threshold 0.7, 16 terms, 120 pairs; (**B**) Figure 2A, threshold 0.5, 33 terms, 528 pairs; and (**C**) Figure 2B, threshold 0.8, 54 terms, 1431 pairs. The pairwise semantic similarities for all terms are used to generate the two-dimensional transformation for the scatterplot. Thus terms with higher semantic similarities should be placed closer to each other (shorter distances) on the scatterplot. Comparing semantic similarities (SemSim) and scatterplot distances (Distance) demonstrates the effectiveness of the two-dimensional transformation procedure to optimise the positioning of the terms on the scatterplot. Semantic similarities computed by GO-Figure! as described in the main text. Scatterplot distances computed from the x and y coordinates from the multidimensional scaling procedure described in the main text. Regression lines plotted using geom_smooth(method="auto", level=0.75) with ggplot in R.

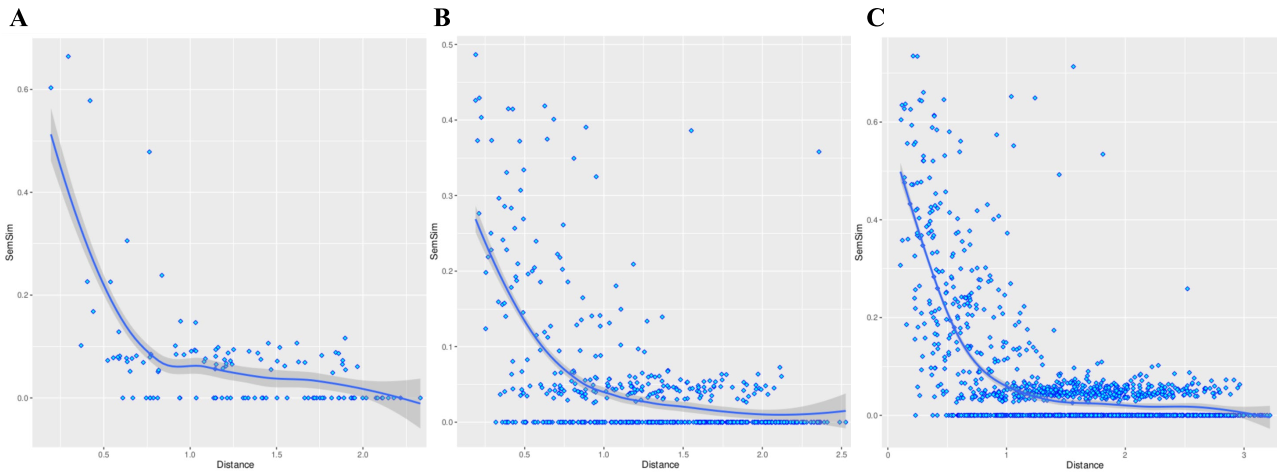

Figure S3. Within group and between group scatterplot distances averaged for all pairs of Gene Ontology terms in groups built using a range of semantic similarity thresholds using the dataset from main text Figure 1. The thresholds vary from stringent (0.9) where only the most similar terms are grouped together, to relaxed (0.1) where larger sets of less closely related terms can be grouped together (y-axis). Scatterplot distances are computed from the x and y coordinates from the multidimensional scaling procedure described in the main text (x-axis). Singleton terms, i.e. those that are not grouped during redundancy reduction, are excluded from these comparisons. The average distances amongst terms within groups (green) increases expectedly as the semantic similarity threshold is relaxed because more distantly related terms are grouped together. The average distances amongst terms between groups (purple) changes little across different semantic similarity thresholds, as the dimensionality reduction aims to always make use of the maximum amount of two-dimensional space. These distances are nevertheless slightly smaller at the highest stringencies because there are more groups to position on the scatterplot and hence overall they are closer together on average. Thus GO term redundancy reduction (grouping) brings together terms that are close in two-dimensional space as defined through dimensionality reduction applied to all pairwise semantic similarity scores. In addition, average distances to terms outside a given group are substantially larger, even at low semantic similarity thresholds, indicating that dissimilar terms are generally assigned to distinct groups.

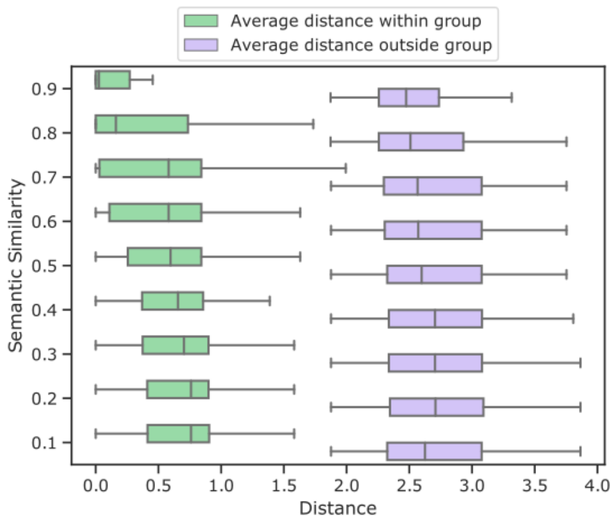

Figure S4. Terms and groups of terms shown on a subset of the Gene Ontology Biological Process graph using the 46 terms from main text Figure 1. The GO terms that are part of the three largest groups in Figure 1A (see lists in Supplementary Table S1) are indicated with coloured arrows. Yellow arrows show terms belonging to group 1, which is represented by the term ‘cell cycle process’. All terms in this group are related to organisational processes prior to the mitotic process, including spindle localization and chromosome condensation. Blue arrows show terms belonging to group 7, which is represented by the term ‘regulation of cell cycle’. All terms in this group are terms representing regulatory and checkpoint processes of the mitotic cell cycle. Pink arrows show terms belonging to group 9, which is represented by the term ‘microtubule-based processes’. All terms in this group are related to microtubule organisation. The graph was drawn using the GOView web-server from http://www.webgestalt.org/ with groups of terms subsequently manually highlighted with different coloured arrows.

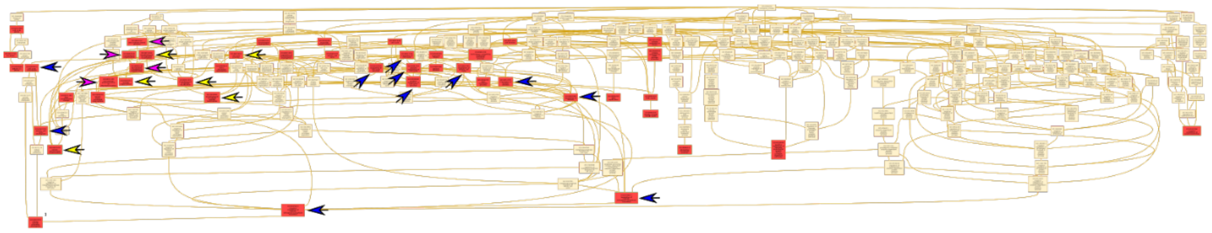

Figure S5. GO-Figure! summary visualisation of a list of 127 Gene Ontology terms found to be enriched amongst hypermethylated genes in subcutaneous adipose tissue after sleep deprivation (from Cedernaes *et al*. 2018, DOI: 10.1126/sciadv.aar8590). The authors noted that "hypermethylated genes were found to enrich for biological pathways such as lipid response and cell differentiation". The summary reduces the term list to 25 representatives with term groupings presented in Table S5. The plot highlights 'response to lipid' (group #3, 16 terms) and 'cell morphogenesis involved in differentiation' (group #4, 7 terms). It further points to 'nervous system process' (group #1, 21 terms, with many sensory perception and signalling processes) and 'positive regulation of synaptic transmission' (group #2, 20 terms, dominated by regulation of signalling processes), suggesting that roles in signalling could also be a key biological feature of this set of hypermethylated genes. Plotted with options: -i hyper.txt -w HYPER -m 30 -t 'Hypermethylated' -b 0.55 -f small -p plasma -si 0.5 -n bpo -o HYPER -s pval -c members.

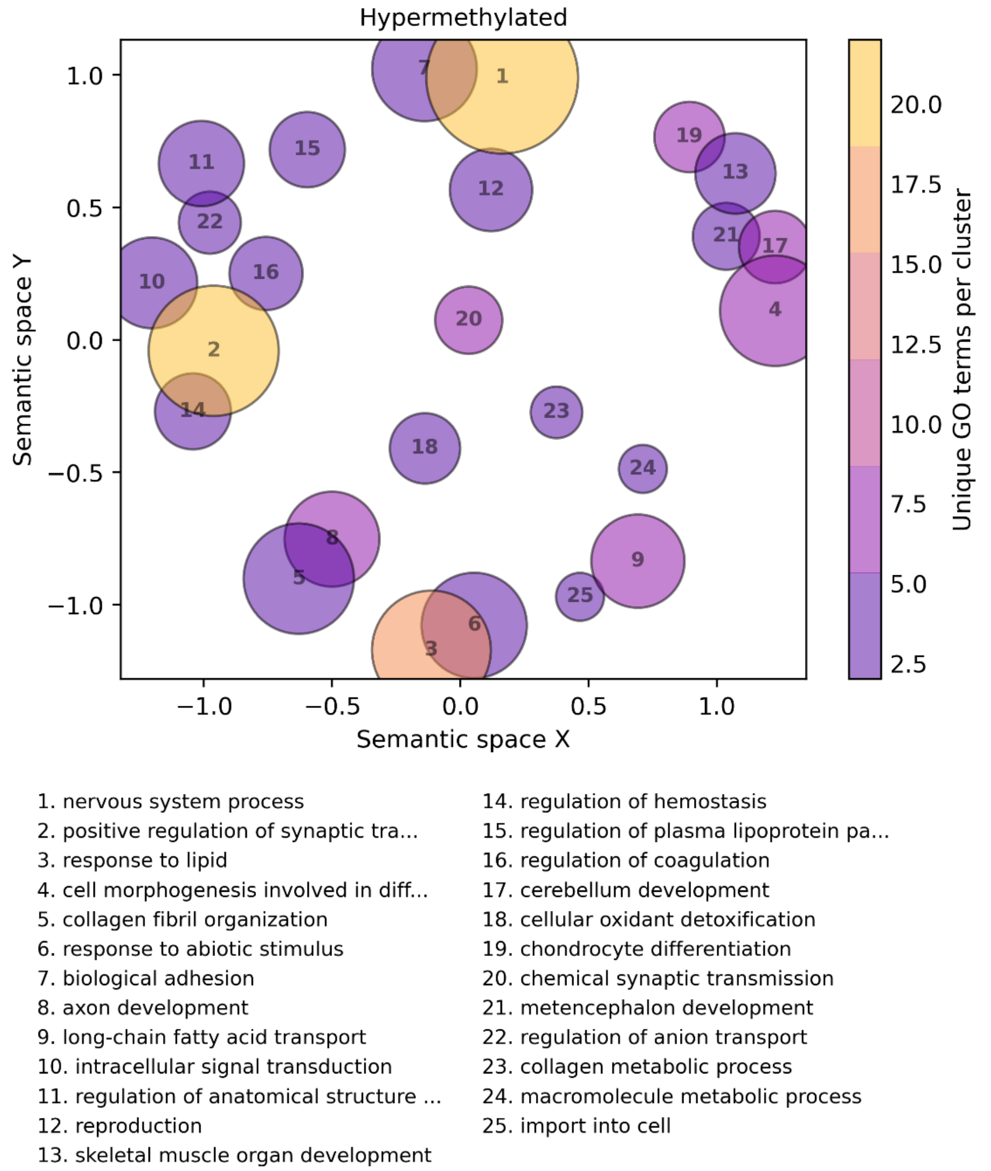

Figure S6. GO-Figure! summary visualisation of a list of 108 Gene Ontology terms found to be enriched amongst hypomethylated genes in subcutaneous adipose tissue after sleep deprivation (from Cedernaes *et al*. 2018, DOI: 10.1126/sciadv.aar8590). The authors noted that "hypomethylated genes were related to pathways such as DNA damage response regulation and lipid metabolism". The summary reduces the term list to 25 representatives with term groupings presented in Table S6. The plot highlights 'positive regulation of DNA damage response' (group #1, 22 terms) and 'regulation of fatty acid metabolic process' (group #6, 9 terms). It further points to 'cartilage development' (group #2, 11 terms) and 'skeletal system morphogenesis' (group #3, 5 terms), suggesting that roles in skeletal muscle wasting could also be a key biological feature of this set of hypomethylated genes. Plotted with options: -i hypo.txt -w HYPO -m 30 -t 'Hypomethylated' -b 0.55 -f small -p plasma -si 0.5 -n bpo -o HYPO -s pval -c members.

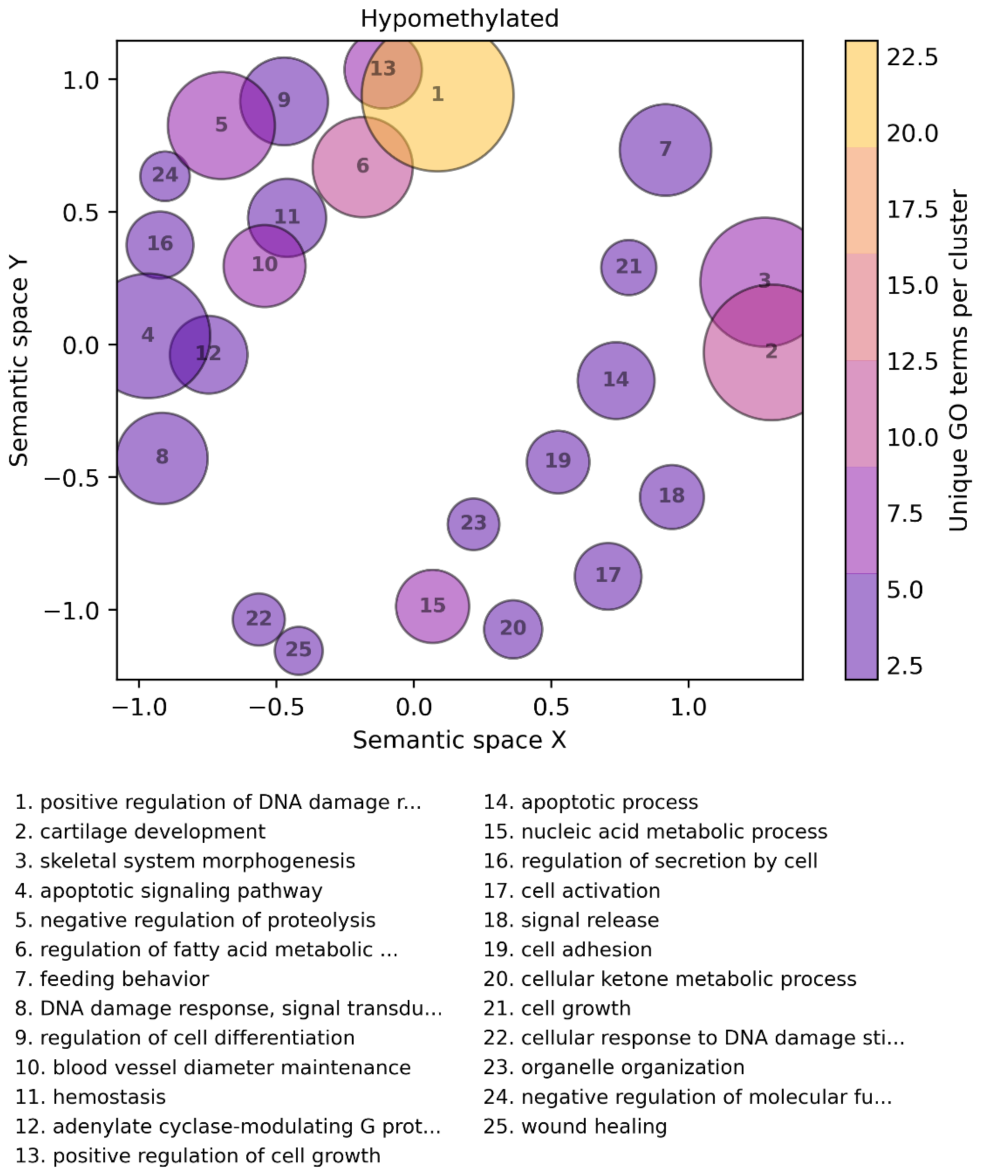

Supplement: Supplementary file 1 [file Data_Sheet_1.PDF]
